# Complement‐mediated vascular inflammation contributes to anti‐amyloid mAb‐induced ARIA in mice

**DOI:** 10.1002/alz70861_108870

**Published:** 2025-12-23

**Authors:** Praveen Bathini, Ilina Navani, Cynthia A Lemere

**Affiliations:** ^1^ Ann Romney Center for Neurologic Diseases, Brigham and Women's Hospital; Harvard Medical School, Boston, MA USA; ^2^ Harvard Medical School, Boston, MA USA; ^3^ Ann Romney Center for Neurologic Diseases, Brigham and Women's Hospital, Boston, MA USA; ^4^ Ann Romney Center for Neurologic Diseases, Brigham and Women's Hospital, Harvard Medical School, Boston, MA USA

## Abstract

**Background:**

Anti‐amyloid antibody immunotherapy in Alzheimer’s disease (AD) has been associated with amyloid‐related imaging abnormalities (ARIA) with vasogenic edema and microhemorrhages in the brain, especially in ApoE4 carriers. In this study, we examined whether treatment with a murine version of bapineuzumab (3D6‐L) leads to microhemorrhages and investigated potential complement and immune cell activation, using different treatment timelines in AD mouse models.

**Method:**

In an acute study, 25‐month‐old APP NL‐G‐F mice were immunized with 3D6‐L or IgG2a control on Day 1 and Day 4, and euthanized on Day 7 to assess early antibody binding, classical complement pathway (CCP) activation, and immune cell recruitment. In addition, aged 16‐17‐month‐old APP/PS1dE9; huApoE4 mice received weekly 3D6‐L or IgG2a for 7 or 13 weeks. Amyloid burden, complement proteins, and vascular integrity were assessed by ELISA and immunostaining. Microhemorrhages (MH) were evaluated with Prussian blue, and early RBC extravasation by H&E. Differential gene expression was analyzed by brain RNAseq. Brain sections were stained for perivascular macrophages (PVMs), fibroblasts, T and B‐cells, and neutrophils and analysis is ongoing.

**Result:**

In the acute study, 3D6‐L preferentially bound vascular amyloid and initiated early CCP. Although T‐cells and PVMs immunoreactivity (IR) showed no significant group differences, an upward trend in Trem2 IR was observed. In the 7‐week treatment study, we detected increased complement activation, RBC extravasation, and MHs. RNAseq revealed upregulation of complement genes. CD3+ and CD8+ T cells were detected near the vasculature, suggesting complement anaphylatoxin‐mediated immune infiltration. In the 13‐week study, 3D6‐L reduced insoluble Aβ but increased brain MHs, and C3 and C5b‐9 IR. We observed increased C3‐associated PVMs, ApoE/fibroblast colocalization, and BBB leakage. The classical complement pathway was fully activated. Gene expression analysis showed upregulation of inflammation and complement‐related genes in the brain. Immune cell expression analysis is underway.

**Conclusion:**

Anti‐amyloid antibodies initially bind vascular amyloid and trigger complement activation. Sustained treatment promotes C3 and C5b‐9 accumulation, RBC leakage, and recruitment of peripheral immune cells to the BBB; driven potentially by C3a/C5a signaling. These results highlight complement and vascular immune cell changes as potential early indicators of ARIA and as therapeutic targets. Funding: NIH NINDS 1R01NS136122‐01 (CAL).

